# The PFP and ESG protein function prediction methods in 2014: effect of database updates and ensemble approaches

**DOI:** 10.1186/s13742-015-0083-4

**Published:** 2015-09-14

**Authors:** Ishita K. Khan, Qing Wei, Samuel Chapman, Dukka B. KC, Daisuke Kihara

**Affiliations:** 1 Department of Computer Sciences, Purdue University, West Lafayette, IN 47907 USA; 2 Department of Biological Sciences, Purdue University, West Lafayette, IN 47907 USA; 3 Department of Computational Science and Engineering, North Carolina A & T State University, Greensboro, NC 27411 USA

**Keywords:** Protein function, sequence, CAFA, function prediction, PFP, ESG, consensus method, ensemble method, gene annotation

## Abstract

**Background:**

Functional annotation of novel proteins is one of the central problems in bioinformatics. With the ever-increasing development of genome sequencing technologies, more and more sequence information is becoming available to analyze and annotate. To achieve fast and automatic function annotation, many computational (automated) function prediction (AFP) methods have been developed. To objectively evaluate the performance of such methods on a large scale, community-wide assessment experiments have been conducted. The second round of the Critical Assessment of Function Annotation (CAFA) experiment was held in 2013–2014. Evaluation of participating groups was reported in a special interest group meeting at the Intelligent Systems in Molecular Biology (ISMB) conference in Boston in 2014. Our group participated in both CAFA1 and CAFA2 using multiple, in-house AFP methods. Here, we report benchmark results of our methods obtained in the course of preparation for CAFA2 prior to submitting function predictions for CAFA2 targets.

**Results:**

For CAFA2, we updated the annotation databases used by our methods, protein function prediction (PFP) and extended similarity group (ESG), and benchmarked their function prediction performances using the original (older) and updated databases. Performance evaluation for PFP with different settings and ESG are discussed. We also developed two ensemble methods that combine function predictions from six independent, sequence-based AFP methods. We further analyzed the performances of our prediction methods by enriching the predictions with prior distribution of gene ontology (GO) terms. Examples of predictions by the ensemble methods are discussed.

**Conclusions:**

Updating the annotation database was successful, improving the F_max_ prediction accuracy score for both PFP and ESG. Adding the prior distribution of GO terms did not make much improvement. Both of the ensemble methods we developed improved the average F_max_ score over all individual component methods except for ESG. Our benchmark results will not only complement the overall assessment that will be done by the CAFA organizers, but also help elucidate the predictive powers of sequence-based function prediction methods in general.

**Electronic supplementary material:**

The online version of this article (doi:10.1186/s13742-015-0083-4) contains supplementary material, which is available to authorized users.

## Background

Advancement in high-throughput genome sequencing technologies in the last decade has posed a challenge in the arena of protein bioinformatics - the exponential growth of new sequence data that awaits functional elucidation. To achieve fast and automatic function annotation of novel/nonannotated proteins, a large variety of automated function prediction (AFP) methods have been developed. Conventional protein function prediction methods such as BLAST [1], FASTA [2], and SSEARCH [3] rely on the concept of homology. There are also prediction methods based on motif/domain searches, such as PRINTS [4], ProDom [5], PFAM [6], BLOCKS [7], and integrative methods that are based on some of the above-mentioned resources, such as InterPro [8]. In addition, there are several methods that thoroughly extract function information from sequence database search results using different strategies. These methods include GOFigure [9], OntoBlast [10], Gotcha [11], GOPET [12], the protein function prediction (PFP) method [13, 14], ConFunc [15], and the extended similarity group (ESG) method [16]. Three methods, SIFTER [17], FlowerPower [18], and Orthostrapper [19], use phylogenetic trees to transfer functions to target genes in the evolutionary context. There are other function prediction methods that consider coexpression patterns of genes [20–24], 3D structures of proteins [25–34], and interacting proteins in large-scale protein-protein interaction networks [35–40].

To evaluate the function prediction performances of AFP methods on a large scale, the Critical Assessment of Function Annotation (CAFA) was developed as a community-wide experiment [41]. In CAFA, participants submit function annotation using gene ontology (GO) [42, 43] terms for a large number of target proteins. The organizers evaluate the accuracy of predicted GO terms for a subset of target annotations that are newly revealed after the submission deadline. In the second round of CAFA, i.e. CAFA2, for which an evaluation meeting was held as a special interest group meeting at the 2014 Intelligent Systems in Molecular Biology (ISMB) conference in Boston, a total of 100,816 target protein sequences from 27 species were provided. Compared with CAFA1 (48,298 targets in 18 species) that was held in 2001, CAFA2 had approximately twice as many targets.

We have participated in CAFA1 and CAFA2 with two of our methods, PFP [13, 14] and ESG [16]. PFP extends PSI-BLAST [1] search by extracting and scoring GO annotations taken from distantly similar sequences and applies contextual associations of GO terms to primarily enhance sensitivity of function prediction [13, 14]. PFP was ranked highest in the function prediction category in the Critical Assessment of techniques for protein Structure Prediction (CASP) [44]. ESG performs iterative sequence database searches and assigns probability scores to GO terms based on their relative similarity scores to multiple-level neighbours in a protein similarity graph [16]. In the CAFA1 experiment, ESG was ranked fourth in the molecular function (MF) GO category among 54 participating groups [41].

In this work, we report benchmark results and enhancements made to PFP [13, 14] and ESG [16] as preparation for the CAFA2 experiment, prior to participation. We first discuss the effect of updated annotation databases that are used in PFP and ESG. The annotation databases for PFP and ESG have not been updated since 2008, when the two methods were initially developed. In this study, we also wanted to examine the improved methods for predicting the current GO annotations of protein sequences by using the updated databases.

Next, we constructed two ensemble function prediction methods, consensus method (CONS) and frequent pattern mining (FPM), that combine GO predictions from PFP [13, 14], ESG [16], PSI-BLAST [1], PFAM [6], FFPred [45], and HHblits [46]. Among the six individual methods, ESG with the updated database performed the best. Both CONS and FPM showed improvement in the average F_max_ score as compared with all the individual component methods except the ESG method. Successful and unsuccessful cases of the CONS ensemble method are discussed.

### Data description

The benchmark dataset consists of 2,055 nonredundant query protein sequences selected from the UniProt Reference Clusters (UniRef) database [47] (version 30/07/2013). UniRef provides clustered sets of sequences from the UniProt knowledgebase. We selected a cluster resolution of 50 % sequence identity. Among these UniRef50 clusters, we selected one representative protein from each of the clusters that satisfied the following two criteria: 1) each cluster representative should have at least 1,500 proteins in its cluster, and 2) the cluster representative protein should have a nonempty GO term annotation in UniProt. We ran the function prediction methods for sequences in this benchmark dataset and evaluated the method's prediction performances.

### Analyses

#### Database update for PFP and ESG

First we discuss the effect of updating the underlying databases of PFP and ESG. The framework of both methods consists of three steps: 1) retrieving similar sequences to a query sequence from a sequence database, 2) extracting GO terms that are associated with the retrieved sequences, and 3) predicting GO terms for the query (see Methods). Two different databases are used in the procedure: a sequence database for Step 1, against which the query is searched, and a second database for Step 2 that stores GO terms for the retrieved sequences. The latter database is referred to as the annotation database.

The sequence database that is searched against (Step 1) for both PFP and ESG is UniProt (the Swiss-Prot portion). This database is referred to as Swiss-Prot-SeqDB. We have been using a 2008 version of Swiss-Prot, but this time it was updated to the version 20 January 2013.

PFP and ESG use different annotation databases (Step 2). PFP uses the so-called PFPDB, which is an integrated database of GO terms taken from multiple databases. PFPDB is discussed in detail later in this section. ESG uses the GO database downloaded from the website of the Gene Ontology Consortium as its annotation database. The previous version is from 2008, and the new version used in this work (and in CAFA2) was downloaded in 2013.

Table 1 describes the differences in the number of sequences and GO terms between the old and new databases. The number of sequences in Swiss-Prot-SeqDB is expanded in the new database to more than double the size (2.45 times) of the old database.

**Table 1:** Database update

	2008 version	2013 version
Sequence database (Swiss-Prot-SeqDB)		
Number of sequences	211,104	514,673
PFPDB (Annotation database for PFP)		
Number of unique GO terms	18,327	35,029
External resources for PFPDB	HAMAP, InterPro, Swiss-Prot-keywords, PFAM, PRINTS, ProDom, PROSITE, SMART, TIGRFam	HAMAP, InterPro, PFAM, PRINTS, ProDom, PROSITE, SMART, TIGRFam, PIRSF, Reactome
Annotation database for ESG		
Number of GO terms	13,420	23,896

Table 1 also contains data for PFPDB, the annotation database used for PFP. PFPDB is a collection of GO terms from multiple annotation resources, including UniProt-Swiss-Prot. The updated PFPDB database did not include annotations from Swiss-Prot keywords and added two new annotation resources to the previous ones (PIRSF [48] and Reactome [49]). With the updated PFPDB, the functional association matrix (FAM), which is the conditional probability *P*(*f*_*a*_|*f*_*i*_) in Equation 1 (in the Methods section) used in PFP, was also updated. In PFPDB, the total number of GO terms in the updated database is increased to almost double (1.91 times) the number from the old database. The number of unique GO terms in the annotation database for ESG, which is the GO database, is increased by 1.78 times from 2008–2013.

In Table 2, we show the effects of combining multiple annotation resources (from which annotations are transferred) for the updated PFPDB in terms of the sequence coverage and the GO coverage. The sequence coverage is the percentage of sequences in Swiss-Prot that have at least one GO term annotation. The GO coverage is the percentage of GO terms that are included in PFPDB relative to the entire GO vocabulary. Having a large coverage is essential for the PFP and ESG function prediction methods, because it directly affects the algorithms’ ability to retrieve function information from a PSI-BLAST search result.

**Table 2:** Coverage from additional resources in updated PFPDB

	Sequence coverage (%)*	GO coverage (%)^†^
Swiss-Prot-GO	94.50	60.27
HAMAP	58.35	3.55
InterPro	95.75	10.59
PFAM	92.34	6.47
PRINTS	22.26	3.09
ProDom	5.39	1.18
ProSite	56.45	2.53
SMART	23.25	1.26
TIGRFam	49.92	4.78
PIRSF	18.38	4.29
Reactome	1.46	0.01
ALL	98.42	60.83

*Sequence coverage is the percentage of sequences in Swiss-Prot annotated with at least one GO term after addition of translated terms from the format in column 1. ^†^GO coverage is the percentage of terms in the GO vocabulary represented in Swiss-Prot after addition of translated terms from the resource in column 1

Each of the Swiss-Prot-GO, InterPro, and PFAM databases has very high (>90 %) sequence coverage as an annotation resource. In terms of the GO coverage, Swiss-Prot-GO has the highest percentage. The rest of the databases have relatively low coverage, with InterPro being the highest among them; however, its GO coverage is as low as 10.59 %. Overall, 98.42 % of Swiss-Prot sequences have at least one GO annotation, and 60.83 % of GO terms in the current GO vocabulary are represented in PFPDB. Compared with the sequence and GO coverage of Swiss-Prot-GO, which was the starting point of the annotation, adding more GO terms from additional sources did not gain much coverage, only about 4 % for the sequence coverage and 0.5 % for the GO coverage. These results are substantially different from when we constructed PFPDB originally in 2008 [14]. At that time, the sequence coverage jumped from 13.4 to 92.9 % by importing GO terms from the additional sources [14] (Table 2). The reason for the small gain in coverage can probably be attributed to the fact that GO annotations in Swiss-Prot have been far better developed since then, and annotations in different databases are now better shared between databases.

#### Benchmarking prediction accuracy of PFP and ESG

**Fig. 1:**
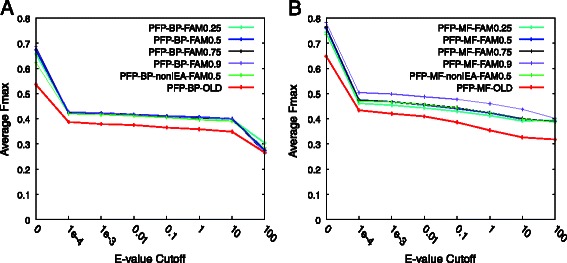
Performance of protein function prediction (PFP) evaluated on GO terms including parental terms. Performance of PFP using the new and the old PFP database (PFPDB). Before evaluating predictions, both predicted and true GO terms were propagated to the root of the ontology. (**a**) Evaluation on biological process (BP) GO terms. (**b**) Evaluation on molecular function (MF) GO terms

Figure 1 shows the results of PFP using the old and the updated PFPDB. To simulate a realistic scenario in which close homologs of a query do not exist in the sequence database, sequences similar to the target in the sequence database that have a certain E-value or smaller (i.e. more significant) were removed. The E-value cut-off is shown along the *x*-axis of the figure. Thus, for example, with an E-value of 0.01 (shown by *x* = 0.01 in the figure), all the sequences in the database that have an E-value of 0.01 or smaller to the query were removed. At *x* = 0, sequence hits with an E-value of 0 were removed in order to avoid annotation transfer from exactly matched sequences. The *y*-axis reports the average F_max_ score (See Methods Section) over all benchmark targets.

For this evaluation, we extend both predicted and true GO terms of each target with parental GO terms in the GO hierarchy. For a predicted or true GO term GO^i^, all parental GO terms of GO^i^ in the GO hierarchy (more precisely, a directed acyclic graph or DAG) were added, and the performance evaluation was done by comparing the extended GO term sets. This parental propagation on the true and predicted annotation sets was also adopted in the official CAFA assessments. The performance evaluation without applying the parental propagation is provided in Figures S1 and S2 in Additional file 1.

For PFP with the updated PFPDB, different functional association matrix (FAM) score cut-offs were tested. The FAM score is the probability that a GO term *f*_*a*_ coexists in the annotation of a protein when another GO term *f*_*i*_ already exists in the annotation of the protein. Concretely, it is the conditional probability *P*(*f*_*a*_|*f*_*i*_) in Equation 1 in the Methods section. For example, in Fig. 1, PFP-BP(or MF)-FAM0.9 represents the prediction results of PFP using the updated PFPDB and only very strongly associated GO terms in FAM, with a FAM score of 0.9 or higher. On the other hand, PFP-BP(or MF)-FAM0.25 used many GO term associations, including ones that are weakly associated, with a conditional probability of 0.25 or higher. For more details of the FAM score, refer to the original paper describing the PFP algorithm [13, 14].

Figure 1 shows predictions for the biological process (BP) GO category (Fig. 1a) and for the molecular function (MF) GO category (Fig. 1b), separately. In Fig. 1a, all of the PFP predictions with the new PFPDB performed better than PFP with the old database (PFP-BP-OLD). For PFP-BP/MF-OLD, a FAM score threshold of 0.9 was used. Among five different FAM score threshold values (0.25–0.9), PFP-BP-FAM0.9 showed the largest average F_max_ accuracy across all the E-value cut-off scores. At the first E-value cut-off, 0.0, PFP-BP-FAM0.9 achieved the largest average F_max_ score of 0.6873, and PFP-BP-FAM0.75 showed the second highest score of 0.6856.

Comparing results using the full PFPDB (PFP-BP-FAM0.5) and those using a subset of GO terms in PFPDB that have experimental evidence (i.e. GO terms that are not inferred from electronic annotation, non-IEA; PFP-BP-nonIEA-FAM0.5), the former had a larger average F_max_ score, as shown in Fig. 1a/b. In Fig. 1 we excluded IEA GO terms only from PFPDB and kept IEA GO terms for the target proteins as correct terms. We also evaluated predictions when IEA GO terms are excluded from correct GO terms in the benchmark dataset (Figure S3 in Additional file 1), where a substantial drop in the accuracy was observed. This is because the IEA GO terms of target proteins, which can be easily identified by sequence similarity, are now considered to be false positives.

Figure 1b shows the performance on MF GO terms. Overall, prediction accuracy for MF (Fig. 1b) was higher than for BP (Fig. 1a). The best-performing prediction setting for MF was again PFP-MF-FAM0.9, with an average F_max_ score of 0.7817 at an E-value cut-off of 0.0, and the second-best performing prediction setting was PFP-MF-FAM0.75 (0.7644). Consistent with Fig. 1a, PFP with the old database was the worst (an F_max_ score of 0.6479 at an E-value cut-off of 0.0). In the original paper of PFP [14], a similar performance comparison was conducted with different FAM score thresholds (Figure 4 in the original paper of PFP [14]), where PFP with a FAM score cut-off of 0.9 was shown to perform best among others. Thus, the findings for the current benchmark with the updated database is consistent with the earlier study [14].

**Fig. 2:**
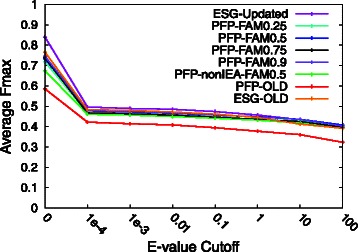
Performance of PFP and extended similarity group (ESG) on GO terms including parental terms. Each predicted and true GO term was propagated to the root of the ontology before evaluation. GO terms in all three ontologies (BP, MF, CC) were used in computing prediction accuracy

In Fig. 2, we added the ESG results to the plots. The F_max_ score was computed using GO terms for all three ontologies (BP, MF, and cellular component [CC]). ESG with the updated database (ESG-Updated) performed the best (average F_max_ score of 0.8401 at an E-value cut-off of 0.0) among the eight settings compared. ESG-OLD was the second best (an average F_max_ score of 0.7655 at an E-value cut-off of 0.0), and PFP-OLD had the lowest accuracy (an average F_max_ score of 0.5852 at an E-value cut-off of 0.0). Similar to Figure S3 in Additional file 1, we removed IEA GO terms from annotation of the benchmark proteins and computed the F_max_ score for all three GO term categories (Figure S4 in Additional file 1), where a similar drop of the F_max_ score was observed.

In summary, updating the databases contributed to improving the prediction accuracy (average F_max_ scores) substantially for both PFP and ESG. ESG showed a higher average F_max_ score than PFP. The best-performing FAM score threshold value for PFP was 0.9, which was consistent with our earlier study.

#### Prediction performance of ensemble methods

Next we discuss the prediction accuracy of two ensemble methods in comparison with individual component methods (Table 3). Two ensemble methods, CONS and FPM, were constructed that combine GO predictions from six individual methods: PFP, ESG, PFAM, PSI-BLAST, HHblits, and FFPred. The CONS method computes a score for a GO term as a weighted sum of scores of the GO terms from the component methods. The weight of a method is prior knowledge of the accuracy of the method. FPM selects combinations of GO terms that are computed from the predictions of multiple methods with a sufficiently high score (see Methods). In Table 3, we show results of two variations of FPM. FPM_maxLen is an FPM method that selects a GO-term set with the largest size (largest number of GO terms) from a candidate pool of predicted GO-term sets with a sufficiently large score. FPM_maxScoreLen, on the other hand, selects the GO-term set with the highest overall score (often resulting in predictions with a small number of GO terms). Overall, out of all the individual and ensemble methods, the most successful method was ESG-Updated, which showed the largest average F_max_ score of 0.8401. CONS had the second highest score (F_max_ score of 0.8085), followed by FPM_maxLen (F_max_ score 0.7937), ESG-Old, and PFP-Updated. On this benchmark, FFPred, PFAM, and HHblits performed very poorly relative to PFP-Updated and ESG-Updated.

**Table 3:** Average F_max_ for individual and ensemble methods

Method	Average F_max_
PFP-Updated	0.7447
PFP-OLD	0.5852
ESG-Updated	0.8401
ESG-OLD	0.7655
FFPred	0.3248
PFAM	0.5583
HHblits	0.4662
PSI-BLAST	0.5991
CONS	0.8085
FPM_MaxLen	0.7937
FPM_MaxScoreLen	0.4628

All true and predicted annotations have been propagated to the root of the ontology. All three GO categories were used in the evaluation

**Fig. 3:**
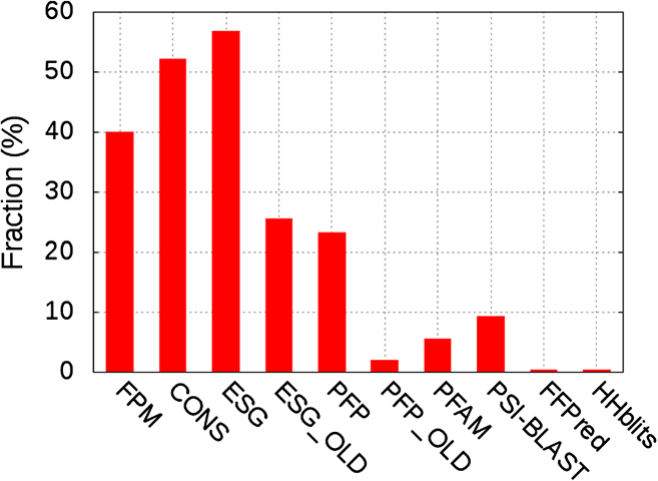
Fraction of queries where each method showed the largest F_max_ score. The fraction on the *y*-axis was computed as the number of queries in which a method had the largest F_max_ score over the total number of queries (2,055 protein sequences). Frequent pattern mining (FPM) in this graph denotes FPM_MaxLen because it performed better than its counterpart, FPM_maxscoreLen. The fraction does not sum up to 100 % because there were cases where multiple methods tied for the largest F_max_ score

To further understand performance of the ensemble methods, we next examined the number of wins for each method, i.e. the number of times that each method showed the largest F_max_ score (Fig. 3). In this analysis, the confidence cut-off values used for each component method were optimized for each target to give the largest F_max_ score to the target; this was done in order to understand how well ensemble methods can assemble individual predictions for the best-case scenario in which each component method offers its best possible prediction. In terms of the number of wins, ESG had the highest, followed by CONS and then FPM, which is consistent with results for the average F_max_ scores (Table 3). Note, there are queries where multiple methods tied for same F_max_ score. Overall, the two ensemble methods did not show better performance than the best component method, ESG, but as illustrated later, there are many cases in which the ensemble methods successfully selected correct GO terms from different component methods.

From Fig. 3, we can see that CONS and FPM provided the most accurate prediction for 52.2 % and 40.0 % of the queries. In addition, Figure S5 in Additional file 1 provides further information about the fraction of queries where predictions from CONS and FPM had the highest, second highest, third highest, etc. F_max_score. It is shown that both CONS and FPM provided the best prediction for the largest fraction of the queries, although many of them were tied with ESG, resulting ESG as the overall best method.

#### Case studies of the CONS method

Table 4 illustrates how CONS combines predictions of the individual methods. The first two examples (Tables 4 and 5) are cases where CONS improved the prediction over the individual methods. Similar to Fig. 3, the F_max_ computation for this analysis is done at the individual protein level. The first example, Table 4, contains predictions for a capsid protein from the Hepatitis E virus (UniProt ID: Q9IVZ8). For this protein, CONS had the highest F_max_ score of 0.667, and PFP had the second highest F_max_ score of 0.575 (F_max_ was computed after parental propagation). In its top hits, CONS correctly predicted all five GO annotations of this protein (shown in bold in the table) together with two parental terms of correct GO terms (shown in italics in the table). Interestingly, PFP, the second-best predictor, predicted only four of the five correct GO terms, whereas the last one, GO:0039615, came from ESG.

**Table 4:** Examples of predictions by CONS and individual-component methods. Capsid protein (UniProt ID: Q9IVZ8)

Method	GO id	Confidence score	GO term
CONS	*GO*:*0019028*	1.00	viral capsid
	**GO:0005198**	0.97	structural molecule activity
	*GO*:*0019012*	0.70	virion
	**GO:0039615**	0.68	T = 1 icosahedral viral capsid
	(GO:0032774)	0.43	
	**GO:0003723**	0.43	RNA binding
	**GO:0044228**	0.43	host cell surface
	**GO:0030430**	0.43	host cell cytoplasm
PFP	**GO:0044228**	1.00	host cell surface
	(GO:0032774)	1.00	
	**GO:0030430**	1.00	host cell cytoplasm
	**GO:0005198**	1.00	structural molecule activity
	**GO:0003723**	1.00	RNA binding
	(GO:0006351)	0.71	
	*GO*:*0043656*	0.65	intracellular region of host
	*GO*:*0033646*	0.65	host intracellular part
	(GO:0008150)	0.59	
	*GO*:*0003676*	0.59	nucleic acid binding
ESG	*GO*:*0019012*	1.00	virion
	*GO*:*0019028*	1.00	viral capsid
	**GO:0039615**	0.99	T = 1 icosahedral viral capsid
	(GO:0019048)	0.15	
	(GO:0030683)	0.15	
	(GO:0039573)	0.15	

GO terms in bold are correct annotations of the protein. Terms in italic indicate parental terms of correct GO terms. Terms in parentheses are wrong predictions

For CONS prediction, GO terms that have a confidence score larger than 0.4 are listed. For PFP prediction, GO terms that have a confidence score larger than 0.5 are listed. For ESG, all predicted GO terms are shown

**Table 5:** Examples of predictions by CONS and individual-component methods. Succinate dehydrogenase iron-sulfur subunit (UniProt ID: P51053)

Method	GO id	Confidence score	GO term
CONS	*GO*:*0051536*	1.00	iron-sulfur cluster binding
	**GO:0009055**	0.25	electron carrier activity
	**GO:0051539**	0.24	4 iron, 4 sulfur cluster binding
	**GO:0046872**	0.24	metal ion binding
	**GO:0006099**	0.22	tricarboxylic acid cycle
	(GO:0016020)	0.21	
	**GO:0051537**	0.21	2 iron, 2 sulfur cluster binding
	**GO:0051538**	0.21	3 iron, 4 sulfur cluster binding
	*GO*:*0016491*	0.16	oxidoreductase activity
	*GO*:*0055114*	0.16	oxidation-reduction process
	*GO*:*0009060*	0.16	aerobic respiration
	**GO:0022900**	0.14	electron transport chain
	(GO:0008177)	0.13	
	…and 9 more terms		
	**GO:0000104**	0.10	succinate dehydrogenase activity
PFP	*GO*:*0055114*	1.00	oxidation-reduction process
	*GO*:*0051540*	1.00	metal cluster binding
	…and 10 more terms		
	**GO:0051539**	0.52	4 iron, 4 sulfur cluster binding
	**GO:0009055**	0.46	electron carrier activity
	(GO:0005886)	0.46	
	(GO:0071944)	0.44	
	(GO:0044435)	0.43	
	**GO:0022900**	0.42	electron transport chain
	…and 9 more terms		
	**GO:0046872**	0.35	metal ion binding
	…and 6 more terms		
	**GO:0006099**	0.33	tricarboxylic acid cycle
	…and 8 more terms		
	**GO:0000104**	0.25	succinate dehydrogenase activity
	(GO:0050136)	0.23	
	(GO:0003954)	0.23	
	**GO:0051537**	0.22	2 iron, 2 sulfur cluster binding
	**GO:0051538**	0.20	3 iron, 4 sulfur cluster binding
ESG	(GO:0005743)	0.66	
	**GO:0006099**	0.66	tricarboxylic acid cycle
	(GO:0008177)	0.66	
	**GO:0009055**	0.66	electron carrier activity
	**GO:0046872**	0.66	metal ion binding
	**GO:0051537**	0.66	2 iron, 2 sulfur cluster binding
	**GO:0051538**	0.66	3 iron, 4 sulfur cluster binding
	**GO:0051539**	0.66	4 iron, 4 sulfur cluster binding
	(GO:0005749)	0.60	
	(GO:0048039)	0.60	
	**GO:0022900**	0.56	electron transport chain
	(GO:0016020)	0.80	
	**GO:0051538**	0.80	3 iron, 4 sulfur cluster binding
	**GO:0051539**	0.80	4 iron, 4 sulfur cluster binding
	*GO*:*0051536*	0.80	iron-sulfur cluster binding
	(GO:0006810)	0.80	
	(GO:0009061)	0.80	
	**GO:0046872**	0.80	metal ion binding
	**GO:0006099**	0.80	tricarboxylic acid cycle
	*GO*:*0009060*	0.80	aerobic respiration
	(GO:0005489)	0.80	
	**GO:0051537**	0.80	2 iron, 2 sulfur cluster binding
	(GO:0005506)	0.80	
	**GO:0000104**	0.80	succinate dehydrogenase activity
	(GO:0006118)	0.80	
	*GO*:*0016491*	0.80	oxidoreductase activity

GO terms in bold are correct annotations of the protein. Terms in italic indicate parental terms of correct GO terms. Terms in parentheses are wrong predictions

For CONS, PFP, and ESG prediction, GO terms that have a confidence score equal to or larger than 0.10, 0.20, and 0.56, respectively, are shown (i.e. up to the last correct GO term). For PSI-BLAST all predicted GO terms are shown

For the second example (Table 5), CONS had the largest F_max_ score of 0.915, followed by PSI-BLAST, which had an F_max_ score of 0.824. The query, succinate dehydrogenase iron-sulfur subunit, has eight GO term annotations. Among them, CONS predicted seven with high confidence scores, and one, GO:0000104, at a low score. Out of these eight GO-term annotations, GO:00051539, GO:0046872, and GO:0006099 were predicted with high scores by three individual methods, PFP, ESG, and PSI-BLAST. GO:0000104 was strongly predicted by PSI-BLAST. GO:0009055 and GO:0022900 were predicted with relatively high scores by ESG and PFP. Thus, CONS can successfully select different correct terms from different methods.

There are also cases showing the opposite trend, where CONS could not improve prediction (Table 6). In the third example, showing the GO annotations of ATP-dependent RNA helicase, the best F_max_ score among the component methods was from ESG (0.761), followed by PSI-BLAST (0.673), PFP (0.667), and PFAM (0.653), while CONS had an F_max_ score of 0.66 and was ranked fourth among all methods. In this example, all five correct GO terms were predicted by ESG, but four of them were with weak scores. PFP predicted only two correct terms, GO:0005524 (ATP binding) with a high score and GO:0000027 (ribosomal large subunit assembly) with a low score, while PSI-BLAST, FFPred, and PFAM only predicted GO:0005524 among the five correct terms. Thus, combining prediction methods could not increase the scores of the correct terms, and rather, introduced over 100 incorrect terms.

**Table 6:** Examples of predictions by CONS and individual-component methods. ATP-dependent RNA helicase SrmB (UniProt ID: P21507)

Method	GO id	Confidence score	GO term
CONS	**GO:0005524**	1.00	ATP binding
	*GO*:*0003676*	0.29	nucleic acid binding
	*GO*:*0004386*	0.24	helicase activity
	*GO*:*0000166*	0.24	nucleotide binding
	*GO*:*0008026*	0.24	ATP-dependent helicase activity
	*GO*:*0016787*	0.20	hydrolase activity
	*GO*:*0003723*	0.19	RNA binding
	(GO:0003677)	0.17	
	…and 37 more terms		
	**GO:0004004**	0.04	ATP-dependent RNA helicase activity
	*GO*:*0044424*	0.04	intracellular part
	(GO:0051716)	0.04	
	(GO:0071843)	0.04	
	…and 142 more terms		
	**GO:0000027**	0.01	ribosomal large subunit assembly
	(GO:0050789)	0.01	
	(GO:0051252)	0.01	
	…and 3 more terms		
	**GO:0033592**	0.01	RNA strand annealing activity
	**GO:0030687**	0.01	preribosome, large subunit precursor
PFP	*GO*:*0044464*	1.00	cell part
	*GO*:*0008150*	1.00	biological process
	*GO*:*0005623*	1.00	cell
	*GO*:*0003676*	1.00	nucleic acid binding
	*GO*:*0004386*	0.99	helicase activity
	*GO*:*0005575*	0.94	cellular component
	*GO*:*0022613*	0.84	ribonucleoprotein complex biogenesis
	*GO*:*0003674*	0.84	molecular function
	(GO:0090304)	0.77	
	*GO*:*0032559*	0.76	adenyl ribonucleotide binding
	**GO:0005524**	0.76	ATP binding
	…and 116 more terms		
	**GO:0004004**	0.11	ATP-dependent RNA helicase activity
	(GO:0080090)	0.10	
	(GO:0070013)	0.10	
	…and 407 more terms		
ESG	**GO:0000027**	0.01	ribosomal large subunit assembly
	*GO*:*0000166*	0.80	nucleotide binding
	*GO*:*0003676*	0.80	nucleic acid binding
	*GO*:*0003723*	0.80	RNA binding
	**GO:0005524**	0.80	ATP binding
	*GO*:*0004386*	0.73	helicase activity
	*GO*:*0008026*	0.73	ATP-dependent helicase activity
	*GO*:*0016787*	0.73	hydrolase activity
	(GO:0000184)	0.46	
	(GO:0005634)	0.46	
	(GO:0006364)	0.46	
	*GO 0042254*	0.46	ribosome biogenesis
	(GO:0005737)	0.38	
	**GO:0004004**	0.28	ATP-dependent RNA helicase activity
	**GO:0000027**	0.07	ribosomal large subunit assembly
	(GO:0005515)	0.07	
	**GO:0030687**	0.07	preribosome, large subunit precursor
	**GO:0033592**	0.07	RNA strand annealing activity

GO terms in bold are correct annotations of the protein. Terms in italic indicate parental terms of correct GO terms. Terms in parentheses are wrong predictions

For CONS prediction, GO terms that have a confidence score equal to or larger than 0.0073 (i.e. up to the last correct GO term) are listed. For PFP prediction, GO terms that have a confidence score equal to or larger than 0.07 are listed. For ESG, all predicted GO terms are shown

#### Adding prior GO term distribution

We have also examined whether the prediction accuracy improves by supplementing a method’s prediction with the known distribution of GO terms in Swiss-Prot. We performed this experiment because it was shown in CAFA1 [41, 50] that the prior distribution itself often has relatively good prediction performance, particularly when no easily identified homologs with known function are available for a query protein. The prior GO-term distribution was added to the predicted GO terms for a target as follows: scores of the predicted GO terms for the target were normalized so that the maximum score became 1.0. In parallel, the frequency (0.0–1.0) of each of the GO terms in Swiss-Prot was determined and normalized so that the most frequently observed GO term had a normalized frequency of 1.0. Then, the top 1,000 most-frequent GO terms in Swiss-Prot were added to the set of predicted GO terms and sorted by the normalized score. The same 1,000 most-frequent GO terms were added to all the targets.

**Fig. 4:**
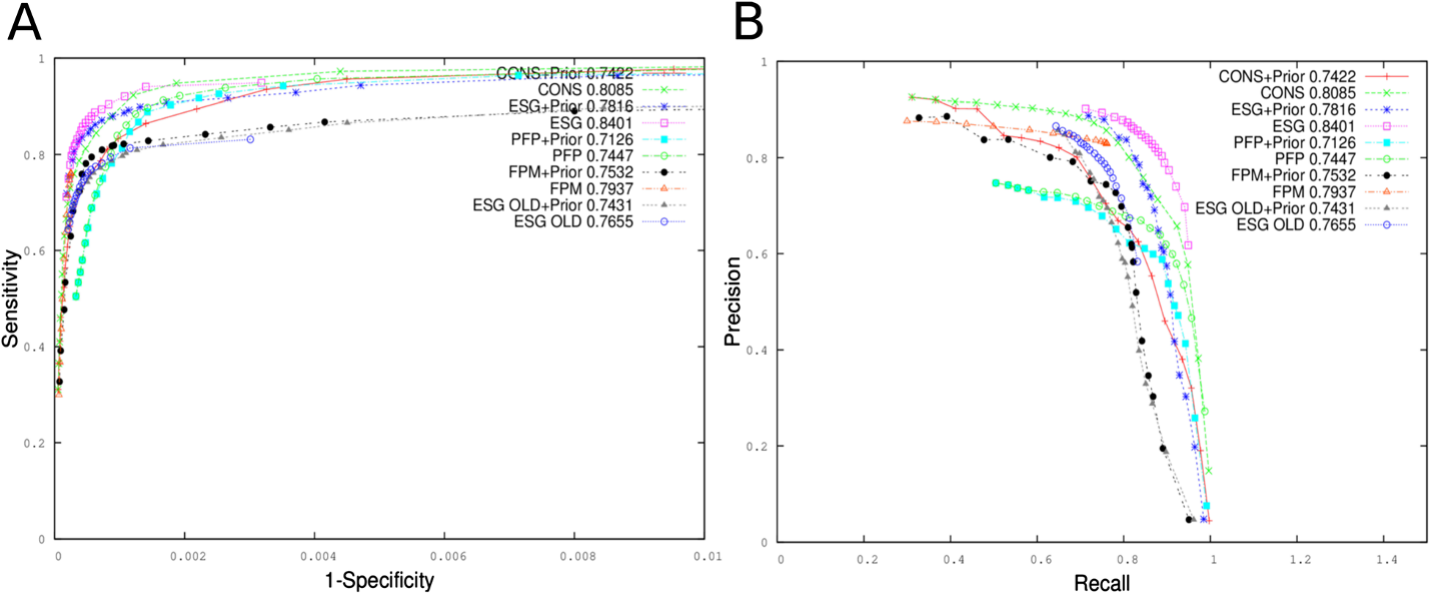
Performance with prior GO term distribution. For PFP, ESG, CONS, FPM, and ESG-OLD, prior GO term distribution was added as a part of the predictions. The numbers shown in the symbol legend are the average F_max_ scores of the methods. (**a**) ROC curve. The *x*-axis is the true negative rate while the y-axis shows the true positive rate. (**b**) The same data are shown in a precision-recall curve

Figure 4 compares the predictions of ESG, PFP, ESG-OLD, and two ensemble methods, CONS and FPM, with and without adding the prior GO distribution. The same data were plotted in two different ways: a receiver-operator characteristics (ROC) curve in Fig. 4a and a precision-recall curve in Fig. 4b. For all the prediction methods, adding prior GO distribution did not improve the accuracy, which can be seen from the plots and the F_max_ values shown in the symbol legends.

## Discussion

We reported a benchmark study of PFP and ESG that has been performed in preparation to participate in CAFA2. An essential task in bioinformatics is to propose and develop new tools and new ideas. However, to support the biological community, it is equally important to maintain and update previously developed software tools so that users can continue using them. For a prediction method, it is important that the prediction accuracy be improved over time so that it can keep pace with other existing methods of the same type. Since the original development of PFP and ESG, the two methods have been benchmarked in CAFA1 by the organizers [41] as well as by our group [50], and their webservers have been recently renovated so that users can obtain prediction information in a more organized fashion [51]. The participation in CAFA2 provided us with a suitable opportunity to update databases for PFP and ESG and to develop ensemble approaches. This article will complement the CAFA2 evaluation paper to be published by the organizers elsewhere in the near future.

We have shown that the prediction performance of PFP and ESG improved by updating databases. Although it may sound obvious to expect better performance with updated databases, it is not necessarily a given, especially considering the recent very-fast expansion of databases. This fast expansion has caused several problems, such as increasing sparseness of useful data (i.e. functional annotation) relative to the size of the sequence databases and the error propagation of incorrect annotations [52]. The comparison between using all annotations and only non-IEA annotations showed that computational annotations are still useful for function prediction; however, more elaborated use of databases might need to be considered if the quality of database information is not maintained throughout the rapid database expansion.

The ensemble methods, CONS and FPM, showed the largest average F_max_ score over all individual component methods except for ESG. The six individual methods used in the ensemble methods may not be the best choice, since their performances were imbalanced, i.e. a large discrepancy in accuracy between PFP/ESG and the rest of the methods. Also, it is noteworthy that all the individual methods use the same source of information as input, i.e. sequence data. Since both CONS and FPM seem to have an ability to assemble the more accurate GO-term set as predictions compared with individual methods (Fig. 3), it will be interesting to apply the two ensemble methods to integrate a better combination of individual methods that use a wide variety of information sources, such as protein structures and protein-protein interaction data and whose performance is more balanced.

Periodic updates and benchmarking of bioinformatics tools is a way for bioinformatics to be an integral part of the biological research community and to be connected to experimental biology. We hope this update report of PFP and ESG helps users to better understand the current status of the tools and will encourage more researchers to use them in research projects.

## Methods

### PFP method

The PFP algorithm [13, 14] uses PSI-BLAST to obtain sequence hits for a target sequence and computes the score for GO term *f*_*a*_ as follows:

1 $$ s\left({f}_{\alpha}\right)={\displaystyle \sum_{i=1}^N{\displaystyle \sum_{j=1}^{Nfunc(i)}\left(\left(- \log \left( Evalue(i)\right)+b\left)P\right({f}_{\alpha}\left|{f}_j\right.\right)\right)}} $$

where *N* is the number of sequence hits considered in the PSI-BLAST hits; *Nfunc*(*i*) is the number of GO annotations for the sequence hit *i*; *E*-*value*(*i*) is the PSI-BLAST E-value for the sequence hit *i*; *f*_*j*_ is the *j*-th annotation of the sequence hit *i*; and constant *b* takes value *2* (= *log*_*10*_*100*) to keep the score positive when retrieved sequences up to an E-value of 100 are used. The conditional probabilities *P*(*f*_*a*_|*f*_*j*_) are used to consider co-occurrence of GO terms in a single sequence annotation, which are computed as the ratio of the number of proteins coannotated with GO terms *f*_*a*_ and *f*_*j*_ as compared with ones annotated only with the term *f*_*j*_. To take into account the hierarchical structure of GO, PFP transfers the raw score to the parental terms by computing the proportion of proteins annotated with *f*_*a*_ relative to all proteins that belong to the parental GO term in the database. The score of a GO term computed as the sum of the directly computed score by Equation 1 and the ones from the parental propagation is called the raw score.

### ESG method

ESG [16] recursively performs PSI-BLAST searches from sequence hits obtained in the initial search from the target sequence, thereby performing multilevel exploration of the sequence-similarity space around the target protein. Each sequence hit in a search is assigned a weight that is computed as the proportion of the -log(E-value) of the sequence relative to the sum of the -log(E-value) from all the sequence hits considered in the search of the same level; this weight is assigned for GO terms annotating the sequence hit. The weights for GO terms found in the second level search are computed in the same fashion. Finally, the score for a GO term is computed as the total weight from the two levels of the searches. The score for each GO term ranges from 0.0 to 1.0.

### FFPred

FFPred [53] predicts more than 440 possible GO terms for a query protein using support vector machines (SVMs) that use more than 200 features of the query. These features are spread among 14 feature types. These types include 20 features describing amino acid composition; seven features describing the sequence itself; 50 features describing the phosphorylation, and others [54]. The SVM-Light [55] package was used to create the SVM classifiers. For each GO term, an SVM classifier was trained by empirically determining the set of kernel parameters and features that performed best in a *k*-fold cross validation of the set of training proteins. The best features were determined on the level of the feature types, so that if the inclusion of the features in a feature type did not improve the SVM, all the features for that feature type were discarded.

#### HHblits

HHblits [46] takes a sequence or multiple sequence alignment as a query and produces a profile hidden Markov model (HMM) from this input. Using the computed HMM, the program iteratively searches a database of profile HMMs, with similar HMMs used to update the query HMM. A prefilter of discretized HMM profiles is used in order to dramatically speed up the process. There are two prefiltering steps when comparing the extended sequence profiles to those of the database. The first makes sure that the score of the largest ungapped alignment between two profiles passes a threshold. Out of the remaining sequences, those with a Smith-Waterman alignment better than the threshold are used. The GO terms from the protein sequences in the final HMM are collected as the predictions of GO terms for the query.

#### CONS

CONS is one of the ensemble methods we constructed that combines predicted GO terms for a target protein from the following six AFP methods, namely, PFP [13, 14], ESG [16], PSI-BLAST [1], PFAM [6], FFPred [53], and HHblits [46]. PSI-BLAST was run for up to three iterations and GO terms were taken from the top five hits. PFAM [56] is a database of HMMs of protein families and domains. A protein can be associated with more than one protein domain HMM. A query sequence was compared with HMMs in PFAM using the HMMER software suite [57] and GO terms were retrieved from hits equal to or below an E-value of 0.01 using the model2GO file associated with PFAM.

CONS combines GO-term predictions from each of the individual methods and provides a consensus confidence score. The consensus confidence score for a GO term is essentially the weighted sum of scores of the GO term from individual methods. The score for GO term *GO*^*i*^ is defined as:

2 $$ CONS\_ score\left(G{O}^i\right)=\frac{{\displaystyle \sum_{m=1}^6{w}_m conf\left(G{O}_m^i\right)}}{{ \max}_{k=1}^N\left( CONS- score\left(G{O}^k\right)\right)} $$

where *m* is an index through each of the six individual methods, and *N* is the total number of unique GO terms for the target predicted by the six methods. The weights *w*_*m*_ reflect prior knowledge of the performances of individual methods *m*, which are the accuracies of the methods (*F*_*max*_ score). *w*_*m*_ for a target sequence was computed on the benchmark dataset after removing the target from the dataset.

#### FPM ensemble method

FPM is a widely used data-mining technique for finding frequently occurring patterns of items. Agrawal et al. [58] first introduced an *a priori* technique of mining all frequent item sets from a transactional database. Later, Tao et al. refined the technique for datasets where each item can have weights [59]. Here we used the flavor of the latter technique to construct an ensemble protein function prediction method from the underlying six individual AFP methods.

We describe the FPM method in the function-prediction setting with a toy example. Let us consider GO-term predictions from three AFP methods for a certain target protein: Method A, B, and C. Let us also assume that each method has a precomputed F_max_ accuracy score: accuracy(Method A) = 0.6, accuracy(Method B) = 0.7, and accuracy(Method C) = 0.5. We assume that the three methods predict GO terms as follows:

- Method A: GO1: 0.5, GO2: 0.6, GO3: 0.4
- Method B: GO2: 0.7, GO3: 0.8, GO4: 0.4, GO5: 0.6
- Method C: GO2: 0.8, GO3: 0.9, GO5: 0.6

Here, GO1:0.5 under “Method A” denotes that Method A predicts GO1 with a confidence score of 0.5.

First, we define two weights that we use throughout the FPM process. *weight*(*m*_*k*_) is a weight given to each method *m*_*k*_ as follows:

3 $$ weight\left({m}_k\right)=\frac{{\displaystyle \sum_{i=1}^{\left|{m}_k\right|} weight\left(G{O}_i\right)}}{\left|{m}_k\right|}\times Accuracy\left({m}_k\right) $$

|m_k_| is the number of GO terms predicted by the method m_k_. Accuracy(m_k_) for a target sequence is computed on the benchmark dataset after removing the target from the dataset.

When the benchmark dataset has multiple target proteins, method weights can be different for each target. For the target in the above toy data,

$$ \begin{array}{l} weight(MethodA)=\frac{0.5+0.6+0.4}{3}\times 0.6=0.3\\ {} weight(MethodB)=\frac{0.7+0.8+0.4+0.6}{4}\times 0.7=0.44\\ {} weight(MethodC)=\frac{0.8+0.9+0.6}{3}\times 0.5=0.38\end{array} $$

*weight*(*GO*_*set*_) is a weight given to a set of GO terms with set size |set| as follows:

4 $$ weight\left(G{O}_{set}\right)=\frac{{\displaystyle \sum_{k=1}^{\left|S\right|} weight\left({m}_k\right)}}{{\displaystyle \sum_{k=1}^{\left|M\right|} weight\left({m}_k\right)}} $$

Here M is the set of all methods, and S is the set of methods that predict GO_set_. For the above toy example, M is 3 and S is 2 for GO2 (since 2 methods, i.e., Method A and Method B, have GO2. GO2 is a GO_set_ of size, |set| = 1). Initially, FPM generates all possible GO_set_s of |set| = 1 and computes the weights of each GO_set_ using Equation 4. In the above toy example, the generated GO_set_s are {GO1, GO2, GO3, GO4, GO5} and the weights are:

$$ \begin{array}{l} weight(GO1)\\ {}=\frac{weight(MethodA)}{weight(MethodA)+ weight(MethodB)+ weight(MethodC)}\\ {}=\frac{0.3}{0.3+0.44+0.38}=0.27\\ {} weight(GO2)=\frac{weight(MethodA)+ weight(MethodB)+ weight(MethodC)}{weight(MethodA)+ weight(MethodB)+ weight(MethodC)}=\frac{1.12}{1.12}=1.0\\ {} weight(GO3)=1.0,\; weight(GO4)=0.39,\; weight(GO5)=0.73\end{array} $$

Then FPM uses a predefined weight cut-off to select the GO_set_s with weights higher than the cut-off and maintains a lexicographic ordering of this selected GO_set_s, L, throughout the rest of the process. In the above toy example, for a weight cut-off of 0.5, FPM selects L = {GO2, GO3, GO5}.

Now, the FPM algorithm runs iteratively starting from |set| = 2 and increases |set| by 1 at each iteration. At each iteration *i*, FPM creates a list, GList_i_ of frequently occurring GO_set_s at the current iteration *i*. At iteration 1, GList_1_ = L. In each iteration *i*, FPM generates a GO_set_ where |set| = i by lexicographically extending each element in GList_i-1_ with each element in set L. FPM then keeps the GO_set_s that have *weight*(*GO*_*set*_) above the weight cut-off and stores them in GList_i_. Iterations continue until no new GO_set_ can be generated. We demonstrate the generation of GList_i_ at each iteration for the above toy example.

- Iteration 1: Candidate GO_set_: {GO1, GO2, GO3, GO4, GO5}, GList_i_: {GO2, GO3, GO5}
- Iteration 2: Candidate GO_set_: {GO2-GO3, GO2-GO5, GO3-GO5}, GList_i_: {GO2-GO3, GO2-GO5, GO3-GO5}
- Iteration 3: Candidate GO_set_: {GO2-GO3-GO5}, GList_i_: {GO2-GO3-GO5}

At iteration *i*, weight(GO_set_) with |set| = i is calculated using Equation 4. In the above list, the weight of GO_set_, GO2-GO5 at iteration 2 is calculated as:

$$ \begin{array}{l} weight\left( GO2- GO5\right)\\ {}=\frac{weight(MethodB)+ weight(MethodC)}{weight(MethodA)+ weight(MethodB)+ weight(MethodC)}\\ {}=\frac{0.44+0.38}{0.3+0.44+0.38}=0.73\end{array} $$

The final result (most frequently occurring GO_set_) is chosen in two ways: FPM_maxLen chooses the maximum-length GO_set_ among all in GList_i_ (for all i), and FPM_maxScoreLen chooses the maximum-length GO_set_ among the highest-scoring GO_set_s in all GList_i_ (among all i). For each target in the benchmark data, the FPM algorithm runs once and generates the most frequently predicted GO terms for that target. We used 0.7 as the predefined weight cut-off.

### F_max_ score

The F_max_ score is computed according to the evaluation strategy taken in CAFA1 [41]. For each target, given a true annotation set T and a predicted annotation set P_t_ from an AFP method above a certain GO confidence score threshold *t*, precision and recall is calculated as follows:

5 $$ \begin{array}{l} precisio{n}_t=\frac{TP}{TP+FP}\\ {} recal{l}_t=\frac{TP}{TP+FN}\end{array} $$

where *TP* = *T* ∩ *P*_*t*_; *FP* = *P*_*t*_\*T*; *FN* = *T*\*P*_*t*_. Then, at each confidence threshold *t*, average precision and recall is calculated across all targets. From these average values, F-measure is calculated as the harmonic mean between precision and recall at each confidence threshold value. Then the maximum F-measure across all thresholds is taken as the F_max_ score:

6 $$ F\; \max =\underset{t}{ \max}\left\{\frac{2* precisio{n}_t* recal{l}_t}{precisio{n}_t+ recal{l}_t}\right\} $$

### Availability of supporting data

Benchmark datasets are hosted in the *GigaScience* GigaDB database [60]. Additional file 1 also contains additional text and Figures S1-S5.

## Additional file

Additional file 1: **Supplemental Material.** **Figure S1.** Performance of PFP evaluated on exact GO terms from BP and MF categories. **Figure S2.** Performance of PFP and ESG evaluated on exact GO terms from all three categories. **Figure S3.** Performance of PFP using IEA and non-IEA GO terms from BP and MF categories. **Figure S4.** Performance of PFP using IEA and non-IEA GO terms of all three GO categories. **Figure S5.** Ranks of CONS and FPM among the benchmarked methods. (DOCX 202 kb)

## Abbreviations

AFP: automated function prediction
BP: biological process
CAFA: Critical Assessment of Function Annotation
CC: cellular component
CONS: consensus method
ESG: extended similarity group
FAM: function association matrix
FPM: frequent pattern mining
GO: gene ontology
MF: molecular function
PFP: protein function prediction

## References

[1] Altschul SF, Madden TL, Schäffer AA, Zhang J, Zhang Z, Miller W (1997). Gapped BLAST and PSI-BLAST: a new generation of protein database search programs. Nucleic Acids Res..

[2] Pearson WR (1990). Rapid and sensitive sequence comparison with FASTP and FASTA. Methods Enzymol..

[3] Pearson WR, Lipman DJ (1988). Improved tools for biological sequence comparison. Proc Natl Acad Sci U S A..

[4] Attwood TK, Bradley P, Flower DR, Gaulton A, Maudling N, Mitchell AL (2003). PRINTS and its automatic supplement, prePRINTS. Nucleic Acids Res..

[5] Bru C, Courcelle E, Carrère S, Beausse Y, Dalmar S, Kahn D. The ProDom database of protein domain families: more emphasis on 3D. Nucleic Acids Res. 2005;212–5. 10.1093/nar/gki034 PMC539988 15608179

[6] Finn RD, Bateman A, Clements J, Coggill P, Eberhardt RY, Eddy SR (2014). The Pfam protein families database. Nucleic Acids Res..

[7] Pietrokovski S, Henikoff JG, Henikoff S (1996). The Blocks database -- a system for protein classification. Nucleic Acids Res..

[8] Hunter S, Jones P, Mitchell A, Apweiler R, Attwood TK, Bateman A (2012). InterPro in 2011: new developments in the family and domain prediction database. Nucleic Acids Res..

[9] Khan S, Situ G, Decker K, Schmidt CJ (2003). GoFigure: Automated Gene Ontology annotation. Bioinformatics.

[10] Zehetner G (2003). OntoBlast function: From sequence similarities directly to potential functional annotations by ontology terms. Nucleic Acids Res..

[11] Martin D, Berriman M, Barton G (2004). GOtcha: a new method for prediction of protein function assessed by the annotation of seven genomes. BMC Bioinformatics..

[12] Vinayagam A, del Val C, Schubert F, Eils R, Glatting KH, Suhai S (2006). GOPET: a tool for automated predictions of Gene Ontology terms. BMC Bioinformatics..

[13] Hawkins T, Luban S, Kihara D (2006). Enhanced automated function prediction using distantly related sequences and contextual association by PFP. Protein Sci..

[14] Hawkins T, Chitale M, Luban S, Kihara D (2009). PFP: Automated prediction of gene ontology functional annotations with confidence scores using protein sequence data. Proteins Struct Funct Bioinf..

[15] Wass MN, Sternberg MJ (2008). ConFunc--functional annotation in the twilight zone. Bioinformatics..

[16] Chitale M, Hawkins T, Park C, Kihara D (2009). ESG: extended similarity group method for automated protein function prediction. Bioinformatics..

[17] Engelhardt BE, Jordan MI, Muratore KE, Brenner SE (2005). Protein molecular function prediction by Bayesian phylogenomics. PLoS Comput Biol..

[18] Krishnamurthy N, Brown D, Sjölander K (2007). FlowerPower: clustering proteins into domain architecture classes for phylogenomic inference of protein function. BMC Evol Biol..

[19] Storm CEV, Sonnhammer ELL (2002). Automated ortholog inference from phylogenetic trees and calculation of orthology reliability. Bioinformatics..

[20] Brown MPS, Grundy WN, Lin D, Cristianini N, Sugnet CW, Furey TS (2000). Knowledge-based analysis of microarray gene expression data by using support vector machines. Proc Natl Acad Sci U S A..

[21] Eisen MB, Spellman PT, Brown PO, Botstein D (1998). Cluster analysis and display of genome-wide expression patterns. Proc Natl Acad Sci U S A..

[22] Gao L, Li X, Guo Z, Zhu M, Li Y, Rao S (2007). Widely predicting specific protein functions based on protein-protein interaction data and gene expression profile. Sci China C Life Sci..

[23] Khatri P, Drâghici S (2005). Ontological analysis of gene expression data: current tools, limitations, and open problems. Bioinformatics..

[24] van Noort V, Snel B, Huynen MA (2003). Predicting gene function by conserved co-expression. Trends Genet..

[25] Gherardini PF, Helmer-Citterich M (2008). Structure-based function prediction: approaches and applications. Brief Funct Genomic Proteomic..

[26] Marti-Renom M, Rossi A, Al-Shahrour F, Davis F, Pieper U, Dopazo J (2007). The AnnoLite and AnnoLyze programs for comparative annotation of protein structures. BMC Bioinformatics..

[27] Martin ACR, Orengo CA, Hutchinson EG, Jones S, Karmirantzou M, Laskowski RA (1998). Protein folds and functions. Structure..

[28] Pal D, Eisenberg D (2005). Inference of protein function from protein structure. Structure..

[29] Ponomarenko JV, Bourne PE, Shindyalov IN (2005). Assigning new GO annotations to protein data bank sequences by combining structure and sequence homology. Proteins Struct Funct Bioinf..

[30] Thornton JM, Todd AE, Milburn D, Borkakoti N, Orengo CA (2000). From structure to function: approaches and limitations. Nat Struct Biol..

[31] Chikhi R, Sael L, Kihara D (2010). Real-time ligand binding pocket database search using local surface descriptors. Proteins Struct Funct Bioinf..

[32] Sael L, Kihara D (2010). Binding ligand prediction for proteins using partial matching of local surface patches. Int J Mol Sci..

[33] Sael L, Chitale M, Kihara D (2012). Structure- and sequence-based function prediction for non-homologous proteins. J Struct Funct Genomics..

[34] Zhu X, Xiong Y, Kihara D (2015). Large-scale binding ligand prediction by improved patch-based method Patch-Surfer2.0. Bioinformatics.

[35] Brun C, Chevenet F, Martin D, Wojcik J, Guenoche A, Jacq B (2003). Functional classification of proteins for the prediction of cellular function from a protein-protein interaction network. Genome Biol..

[36] Chua HN, Sung WK, Wong L (2006). Exploiting indirect neighbours and topological weight to predict protein function from protein-protein interactions. Bioinformatics..

[37] Letovsky S, Kasif S (2003). Predicting protein function from protein/protein interaction data: a probabilistic approach. Bioinformatics..

[38] Nariai N, Kolaczyk ED, Kasif S (2007). Probabilistic protein function prediction from heterogeneous genome-wide data. PLoS One..

[39] Sharan R, Ulitsky I, Shamir R (2007). Network-based prediction of protein function. Mol Syst Biol..

[40] Deng M, Tu Z, Sun F, Chen T (2004). Mapping gene ontology to proteins based on protein-protein interaction data. Bioinformatics..

[41] Radivojac P, Clark WT, Oron TR, Schnoes AM, Wittkop T, Sokolov A (2013). A large-scale evaluation of computational protein function prediction. Nat Meth..

[42] Seok Y, Sondej M, Badawi P, Lewis M, Briggs M, Jaffe H (1997). High affinity binding and allosteric regulation of *Escherichia coli* glycogen phosphorylase by the histidine phosphocarrier protein. HPr. J Biol Chem..

[43] D'Ari L, Rabinowitz J (1991). Purification, characterization, cloning, and amino acid sequence of the bifunctional enzyme 5,10-methylenetetrahydrofolate dehydrogenase/5,10-methenyltetrahydrofolate cyclohydrolase from *Escherichia coli*. J Biol Chem..

[44] Lopez G, Rojas A, Tress M, Valencia A (2007). Assessment of predictions submitted for the CASP7 function prediction category. Proteins Struct Funct Bioinf..

[45] Lobley AE, Nugent T, Orengo CA, Jones DT (2008). FFPred: an integrated feature-based function prediction server for vertebrate proteomes. Nucleic Acids Res..

[46] Remmert M, Biegert A, Hauser A, Söding J (2011). HHblits: lightning-fast iterative protein sequence searching by HMM-HMM alignment. Nat Methods..

[47] UniProt Consortium (2014). Activities at the Universal Protein Resource (UniProt). Nucleic Acids Res.

[48] Wu CH, Nikolskaya A, Huang H, Yeh LS, Natale DA, Vinayaka CR (2004). PIRSF: family classification system at the Protein Information Resource. Nucleic Acids Res..

[49] Joshi-Tope G, Gillespie M, Vastrik I, D'Eustachio P, Schmidt E, de Bono B (2005). Reactome: a knowledgebase of biological pathways. Nucleic Acids Res..

[50] Chitale M, Khan IK, Kihara D (2013). In-depth performance evaluation of PFP and ESG sequence-based function prediction methods in CAFA 2011 experiment. BMC Bioinformatics..

[51] Khan IK, Wei Q, Chitale M, Kihara D (2014). PFP/ESG: automated protein function prediction servers enhanced with Gene Ontology visualization tool. Bioinformatics..

[52] Galperin MY, Koonin EV (1998). Sources of systematic error in functional annotation of genomes: domain rearrangement, non-orthologous gene displacement and operon disruption. In Silico Biol..

[53] Minneci F, Piovesan D, Cozzetto D, Jones DT (2013). FFPred 2.0: improved homology-independent prediction of gene ontology terms for eukaryotic protein sequences. PLoS One..

[54] Lobley A, Swindells MB, Orengo CA, Jones DT (2007). Inferring function using patterns of native disorder in proteins. PLoS Comput Biol..

[55] Joachims T (1999). Making large-scale support vector machine learning practical. Advances in Kernel Methods - Support Vector Learning.

[56] Piatigorsky J (1998). Multifunctional lens crystallins and corneal enzymes. More than meets the eye. Ann N Y Acad Sci.

[57] Breazeale S, Ribeiro A, McClerren A, Raetz C (2005). A formyltransferase required for polymyxin resistance in *Escherichia coli* and the modification of lipid A with 4-amino-4-deoxy-L-arabinose. Identification and function oF UDP-4-deoxy-4-formamido-L-arabinose. J Biol Chem.

[58] Agrawal R, Srikant R. Fast algorithms for mining association rules in large databases. Proceedings of the 20th International Conference on Very Large Data. 1994;487–99.

[59] Tao F, Murtagh F, Farid M. Weighted association rule mining using weighted support and significance framework. Proceedings of the ninth ACM SIGKDD international conference on Knowledge discovery and data mining. 2003;661–6

[60] Ishita K. Khan; Qing Wei; Samuel Chapman; Dukka B. KC; Daisuke Kihara (2015): Supporting data and materials for "The PFP and ESG protein function prediction methods in 2014: effect of database updates and ensemble approaches". GigaScience Database. http://dx.doi.org/10.5524/100161 10.1186/s13742-015-0083-4 PMC4570625 26380077

