# Supplementary material for: The PFP and ESG protein function prediction methods in 2014: effect of database updates and ensemble approaches
Source: Gigascience. 2015 Sep 14;4:43. doi: 10.1186/s13742-015-0083-4 (PMC4570625; doi:10.1186/s13742-015-0083-4)
Supplement: Additional file 1: — Supplemental Material. Figure S1. Performance of PFP evaluated on exact GO terms from BP and MF categories. Figure S2. Performance of PFP and ESG evaluated on exact GO terms from all three categories. Figure S3. Performance of PFP using IEA and non-IEA GO terms from BP and MF categories. Figure S4. Performance of PFP using IEA and non-IEA GO terms of all three GO categories. Figure S5. Ranks of CONS and FPM among the benchmarked methods. (DOCX 202 kb) [file 13742_2015_83_MOESM1_ESM.docx]

Supplemental Material for

# The PFP and ESG protein function prediction methods in 2014: Effect of database updates and ensemble approaches

Ishita K Khan, Qing Wei, Samuel Chapman, Dukka B. K.C. & Daisuke Kihara

Contact: [dkihara@purdue.edu](mailto:dkihara@purdue.edu)

**Figure S1. Performance of PFP evaluated on exact GO terms from BP and MF categories.**

Performance of PFP at eight different E-value cut-offs using new PFPDB (PFP-BP/MF-FAM0.xx) compared with results using the old PFPDB (PFP-BP/MF-OLD). The number in the labels after PFP-BP/MF-FAM is the threshold value of functional association matrix (FAM) score used. PFP-BP/MF-nonIEA-FAM0.5 is PFP predictions using the new PFPDB but only uses non-IEA (Inferred from Electronic Annotation) GO terms with 0.5 for the FAM threshold value. The y-axis is the average F-max score over all benchmark targets. **A**, Evaluation was done on only Biological Process (BP) GO terms. **B**, Evaluation only on Molecular Function (MF) GO terms.

Benchmark results of PFP with the old and the updated PFPDB using the exact GO terms (in predictions and true annotations) without parental propagation are shown in Figure S1. For PFP with the updated PFPDB, different functional association matrix (FAM) score cut-offs were used. Figure S1 shows predictions for the Biological Process (BP) GO category (Figure S1A) and for Molecular Function (MF) GO category (Figure S1B) separately. In Figure 1A, all of the PFP predictions with the new PFPDB performed better than PFP with the old database (PFP-BP-OLD). For PFP-BP/MF-OLD, a FAM threshold of 0.9 was used. Among five different FAM threshold values (0.25 to 0.9) used, PFP-BP-FAM0.9 showed the largest average Fmax accuracy across all the E-value cut-off scores. At the first E-value cut-off, 0.0, PFP-BP-FAM0.9 showed the largest average Fmax score of 0.2912, while PFP-BP-FAM0.75 the second highest score of 0.2864. Comparing the results using the full PFPDB (PFP-BP-FAM0.5) and those using a subset of GO terms in PFPDB that have experimental evidence (i.e. GO terms that are not Inferred from Electronic Annotation, non-IEA) (PFP-BP-nonIEA-FAM0.5), the former had a larger average Fmax score.

Figure 1B is the performance on MF GO terms. Overall, predictions for MF (Figure 1B) were higher than for BP (Figure 1A). The best-performing prediction setting for MF was again PFP-MF-FAM0.9, with an average Fmax score of 0.4089 at an E-value cut-off of 0.0, and PFP-MF-FAM0.75 was the second best (0.396). Consistent with Figure 1A, PFP with the old database was the worst (an Fmax score of 0.2722 at an E-value cut-off of 0.0).

## Figure S2. Performance of PFP and ESG evaluated on exact GO terms from all three categories.

# Performance of PFP and ESG using the old and the new databases. GO terms in all three ontologies (BP, MF, CC) were used in computing Fmax. ESG-Updated and ESG-OLD are prediction sets from ESG using the updated and old databases, respectively.

In Figure S2, we benchmark the prediction accuracy of PFP and ESG using the old and updated databases using the exact GO terms (in predictions and true annotations) without parental propagation. The Fmax score was computed using GO terms for all three ontologies (BP, MF, and Cellular Component (CC)). ESG with the updated database (ESG-Updated) performed the best (average Fmax of 0.5817 at an E-value cut-off of 0.0) among the eight settings compared. ESG-OLD was the second best (an average Fmax of 0.4906 at E-value 0.0), and PFP-OLD has the lowest accuracy (an average Fmax of 0.1919 at E-value 0.0).

**Figure S3.** **Performance of PFP using IEA and non-IEA GO terms from BP and MF categories.** Non-IEA GO annotations both from the PFPDB annotation database and the benchmark dataset are excluded in the non-IEA curve shown in green **A,** evaluation using BP GO terms only; **B,** evaluation using MF GO terms only.

**Figure S4.** **Performance of PFP using IEA and non-IEA GO terms of all three GO categories.** Non-IEA GO annotations both from the PFPDB annotation database and the benchmark dataset are excluded in the non-IEA curve shown in green**.** GO terms from all three ontologies (BP, MF, CC) are used in the evaluation.

In Figure S3-4, we compare the benchmark performances of PFP using IEA and non-IEA annotations. In the non-IEA setting, we use the non-IEA version of the annotation database PFPDB and also the non-IEA version of the true annotations of the benchmark dataset (unlike the non-IEA setting used in the main manuscript, where we included IEA annotations of the benchmark dataset). In Figure S3, we compare and non-IEA benchmark performance of PFP for BP (S3A) and MF (S3B) separately. In both cases, the setting that includes IEA annotations (blue lines, i.e., PFP-BP-FAM0.5 in S3A and PFP-MF-FAM0.5 in S3B) performs significantly better than the IEA versions (green lines in Figure S3A-B). In Figure S4, similar comparison is done by including GO terms from all three ontologies (BP, MF, CC) and further separation between the two (inclusion and exclusion of IEA terms) settings is noticed. The PFP-FAM-0.5 with inclusions of IEA achieves better Fmax score (0.7338).

## Figure S5. Ranks of CONS and FPM among the benchmarked methods.

The fraction of the queries where predictions of **A**, CONS, or **B**, FPM had the largest Fmax score, the second largest, and so on down to the lowest Fmax score. The fraction of CONS-1^st^ and FPM-1^st^ is the same as the value of CONS and FPM in Figure 3. Note that there are cases that multiple methods tied for the largest Fmax score.
